# Plasma biomarkers for diagnosis of Alzheimer's disease and prediction of cognitive decline in individuals with mild cognitive impairment

**DOI:** 10.3389/fneur.2023.1069411

**Published:** 2023-03-02

**Authors:** Pia Kivisäkk, Becky C. Carlyle, Thadryan Sweeney, Bianca A. Trombetta, Kathryn LaCasse, Leena El-Mufti, Idil Tuncali, Lori B. Chibnik, Sudeshna Das, Clemens R. Scherzer, Keith A. Johnson, Bradford C. Dickerson, Teresa Gomez-Isla, Deborah Blacker, Derek H. Oakley, Matthew P. Frosch, Bradley T. Hyman, Anahit Aghvanyan, Pradeepthi Bathala, Christopher Campbell, George Sigal, Martin Stengelin, Steven E. Arnold

**Affiliations:** ^1^Alzheimer's Clinical and Translational Research Unit, Department of Neurology, Massachusetts General Hospital, Harvard Medical School, Boston, MA, United States; ^2^Department of Physiology, Anatomy and Genetics, University of Oxford, Oxford, United Kingdom; ^3^Precision Neurology Program and Center for Advanced Parkinson Research, Harvard Medical School, Brigham and Women's Hospital, Boston, MA, United States; ^4^Department of Neurology, Massachusetts General Hospital, Harvard Medical School, Boston, MA, United States; ^5^Department of Epidemiology, Harvard TH Chan School of Public Health, Boston, MA, United States; ^6^Department of Psychiatry, Massachusetts General Hospital, Harvard Medical School, Boston, MA, United States; ^7^Department of Pathology, Massachusetts General Hospital, Harvard Medical School, Boston, MA, United States; ^8^Meso Scale Diagnostics, LLC., Rockville, MD, United States

**Keywords:** biomarker, plasma, Alzheimer's disease, mild cognitive impairment, pTau181, neurofilament light (NfL), glial fibrillary acidic protein (GFAP), total Tau (tTau)

## Abstract

**Background:**

The last few years have seen major advances in blood biomarkers for Alzheimer's Disease (AD) with the development of ultrasensitive immunoassays, promising to transform how we diagnose, prognose, and track progression of neurodegenerative dementias.

**Methods:**

We evaluated a panel of four novel ultrasensitive electrochemiluminescence (ECL) immunoassays against presumed CNS derived proteins of interest in AD in plasma [phosphorylated-Tau181 (pTau181), total Tau (tTau), neurofilament light (NfL), and glial fibrillary acidic protein (GFAP)]. Two sets of banked plasma samples from the Massachusetts Alzheimer's Disease Research Center's longitudinal cohort study were examined: A longitudinal prognostic sample (*n* = 85) consisting of individuals with mild cognitive impairment (MCI) and 4 years of follow-up and a cross-sectional sample (*n* = 238) consisting of individuals with AD, other neurodegenerative diseases (OND), and normal cognition (CN).

**Results:**

Participants with MCI who progressed to dementia due to probable AD during follow-up had higher baseline plasma concentrations of pTau181, NfL, and GFAP compared to non-progressors. The best prognostic discrimination was observed with pTau181 (AUC = 0.83, 1.7-fold increase) and GFAP (AUC = 0.83, 1.6-fold increase). Participants with autopsy- and/or biomarker verified AD had higher plasma levels of pTau181, tTau and GFAP compared to CN and OND, while NfL was elevated in AD and further increased in OND. The best diagnostic discrimination was observed with pTau181 (AD vs CN: AUC = 0.90, 2-fold increase; AD vs. OND: AUC = 0.84, 1.5-fold increase) but tTau, NfL, and GFAP also showed good discrimination between AD and CN (AUC = 0.81–0.85; 1.5–2.2 fold increase).

**Conclusions:**

These new ultrasensitive ECL plasma assays for pTau181, tTau, NfL, and GFAP demonstrated diagnostic utility for detection of AD. Moreover, the absolute baseline plasma levels of pTau181 and GFAP reflect cognitive decline over the next 4 years, providing prognostic information that may have utility in both clinical practice and clinical trial populations.

## 1. Introduction

Individuals with mild cognitive impairment (MCI) provide a challenge to the clinician due to the difficulty of predicting if an individual will experience further cognitive decline and the rate of decline. We tested the hypothesis that the presence of pathological changes of Alzheimer disease (AD), as indicated by a simple battery of blood tests, might inform that clinical discussion. The recent emergence of ultrasensitive immunoassays for measuring biomarkers for AD has resulted in assays sensitive enough to reliably measure the classic A-T-N biomarkers, which provide the foundation of the current National Institute on Aging and Alzheimer's Association (NIA-AA) research framework for diagnosing AD (1), not only in CSF but also in blood (2), circumventing many of the limitations of the more invasive and/or expensive CSF and PET biomarkers. Various phosphorylated tau (pTau) isoforms, such as pTau181, pTau217, and pTau231, appear thus far to be among the most promising AD biomarkers in plasma (3–7) and have shown promise in predicting progression from MCI to AD dementia in individual patients (6, 8–10). An important next step is to understand and optimize which of these tests are most informative, and to identify analytical platforms to characterize their performance using typical patient derived materials, including historical samples.

In this paper, we evaluated a panel of four novel ultrasensitive electrochemiluminescence (ECL) immunoassays from Meso Scale Diagnostics (MSD; Rockville, MD) of interest in AD: pTau181, total Tau (tTau), neurofilament light (NfL), and glial fibrillary acidic protein (GFAP). We used banked plasma samples from participants in the Massachusetts Alzheimer's Disease Research Center's longitudinal cohort (MADRC-LC) and examined whether we could predict cognitive decline in older individuals with MCI, some of whom had progressed clinically over the next 4 years and some of whom had not. We also used the same assays to evaluate the performance of the four biomarker assays to differentiate the “correct” diagnosis among individuals with an autopsy confirmed, amyloid PET, and/or CSF AD biomarker-based diagnosis of AD, non-AD neurodegenerative diseases (OND), and cognitively normal individuals (CN).

## 2. Materials and methods

### 2.1. Study population

We included a total of 307 participants in the MADRC-LC study, a longitudinal observational study of cognitive aging, AD, and AD-related disorders. Annual assessments include a general and neurological exam, a semi-structured interview to record cognitive symptoms and score the Clinical Dementia Rating scale (CDR Dementia Staging Instrument), a battery of neuropsychological tests (11, 12), and blood collection for all consenting participants. Cognitive status and clinical diagnosis are determined at each visit by a consensus team after a detailed examination and review of all available information according to 2011 NIA-AA diagnostic criteria for MCI (13) and AD (14). APOE genotyping is done on all subjects through the National Alzheimer's Coordinating Center. A subset of participants undergoes imaging and/or CSF biomarker substudies in affiliated protocols and all participants are invited to join a brain donation program.

Eighty five participants had a baseline clinical diagnosis of MCI due to probable AD and a global CDR score of 0.5 (Sample A: Longitudinal prognostic sample). They were subclassified into two groups based on their CDR trajectory over at least five annual follow-up visits over 4 years: MCI-decline (*n* = 47) if their global CDR score increased from 0.5 to ≥1 during follow-up, and MCI-stable (*n* = 38) if there was no change in global CDR score.

Two hundred and thirty eight participants contributed a “high-contrast” diagnostic sample (Sample B) consisting of: a) 95 AD patients with the diagnosis confirmed by intermediate or high AD neuropathologic changes upon autopsy (15), [11C]Pittsburgh Compound-B amyloid PET imaging, and/or CSF biomarkers (1). There were 4.0 ± 2.4 years between plasma collection and death, 0.8 ± 0.8 years between plasma collection and PET imaging, and 2.4 ± 2.8 years between plasma and CSF collection; b) 53 OND participants with a variety of other neurodegenerative diseases and minimal to no AD neuropathological changes on autopsy. There was 2.8 ± 1.9 years between plasma collection and death; and c) 90 cognitively normal controls (CN) with normal neuropsychological testing scores and no subjective cognitive symptoms during 8.8 ± 3.7 years of follow-up.

16 of the participants in the longitudinal sample (A) were also included in the diagnostic sample (B).

### 2.2. Standard protocol approvals, registrations, and patient consents

The study was approved by the Mass General Brigham Institutional Review Board (2006P002104) and all participants or their assigned surrogate decision makers provided written informed consent.

### 2.3. Plasma sampling and analysis

Banked plasma samples collected between 2008 and 2019 were obtained from the Harvard Biomarkers Study Biobank (16). Samples were collected in K_2_EDTA tubes, centrifuged and frozen within 4 h of collection, and stored at −80°C until use. Ultrasensitive MSD S-PLEX^®^ assay kits (MSD, Rockville, MD) employing a sandwich immunoassay format using monoclonal antibodies and ECL detection were used to detect plasma biomarker levels. pTau181 was measured using a now commercial assay (catalog # K151AGMS) following manufacturer's instructions while prototype S-PLEX assays were used for NfL, GFAP and tTau. Calibrators for the different assays were prepared by using recombinant Tau441 expressed in *E. coli*; recombinant phosphorylated tau expressed in a mammalian system and confirmed by mass spectrometry to display phosphorylation at T181; recombinant GFAP expressed in a mammalian system; and bovine NfL purified from spinal cord. Due to the lack of international standards, concentrations of calibrators were assigned *via* biochemical characterization and used to generate a calibration curve for sample quantitation. Lower limit of detection (LLOD) was defined as the concentration that provides a signal 2.5 standard deviations above the mean of the blank. Lower limit of quantification (LLOQ) was defined as the lowest concentration with a coefficient of variation (CV) <20% and a recovery between 80 and 120%. Four quality control (QC) samples spanning the assay range were included in duplicate in each plate for the prototype assays, while one QC sample was included for the commercial pTau181 assay. The samples were codified and randomized so the assay laboratory was blinded to any case information during testing and calculation of concentrations. The samples were distributed over 8 plates per assay, each containing an 8-point calibration curve and QC samples in duplicates and ran over 2 days. Plasma samples were measured as single replicates using 25 uL of undiluted plasma for the NfL and pTau181 assays, or 25 uL of 5-fold diluted plasma for the GFAP and total tau assays. Reported concentrations of GFAP and total tau were corrected for the 5-fold sample dilution.

### 2.4. Statistical analysis

Biomarker concentrations were natural log transformed to satisfy assumptions of normal distribution. Values under LLOQ were assigned the lowest quantifiable value of the assay. All reported *p*-values were adjusted for multiple hypothesis testing using the Benjamini-Hochberg method unless otherwise specified. Differences between diagnostic groups were evaluated using ANOVA adjusting for age, sex, and the biomarker in question followed by Tukey's Honest Significant Difference as the *post-hoc* test. Subgroup analyses between different clinical subsets were performed using logistic regression predicting the subgroup in terms of age, sex, APOE, and the relevant biomarker. To assess classification utility of the biomarkers, area under the curve (AUC) values were computed using logistic regression models as described above (17) and their goodness of fit was assessed using likelihood-ratio test. Effect sizes of each predictor were calculated using Cohen's d. Correlations between markers and with cognitive scores were assessed with Pearson correlation coefficient or Spearman's Rho for ordinal data or distributions containing outlier data. To ameliorate the influence of age on biomarkers, levels were residualized in terms of age before correlative analysis of cognitive scores. The above procedures were carried out using the R statistical software version 4.0.4 (R Foundation for Statistical Computing, Vienna, Austria).

### 2.5. Data availability

Anonymized data not published within this article will be made available by reasonable request from any qualified investigator.

## 3. Results

### 3.1. Analytical performance of assays

All plasma samples had concentrations exceeding LLOD for all four S-PLEX assays (Table 1). The assay signal was linear with concentration across the full calibration range of the assay. The reported LLOD, LLOQ, and ULOQ values for GFAP and tTau were adjusted to account for the 5x dilution used with these assays.

**Table 1:** Assay performance.

	**LLOD**	**LLOQ**	**ULOQ**	**Median conc (Q1-Q3)**	**CV for QC samples**
pTau181 (pg/mL)	0.08	0.46	990	1.7 (1.2–2.6)	8%
tTau (pg/mL)	0.07	0.63	2,000	10.2 (8.2–13.4)	4–7%
NfL (pg/mL)	2.6	9.6	5,300	75 (50–116)	9–13%
GFAP (pg/mL)	8.8	52	10,400	170 (119–228)	5–10%

pTau181, phosphorylated-Tau181; tTau, total Tau; NfL, neurofilament light; GFAP, glial fibrillary acidic protein; LLOD, lower level of detection; L/ULOQ, lower/upper level of quantification; Q1/Q3, Quartile 1/3; CV, coefficient of variation; QC, quality control.

### 3.2. Effects of age and sex on biomarker levels

**Figure 1:**
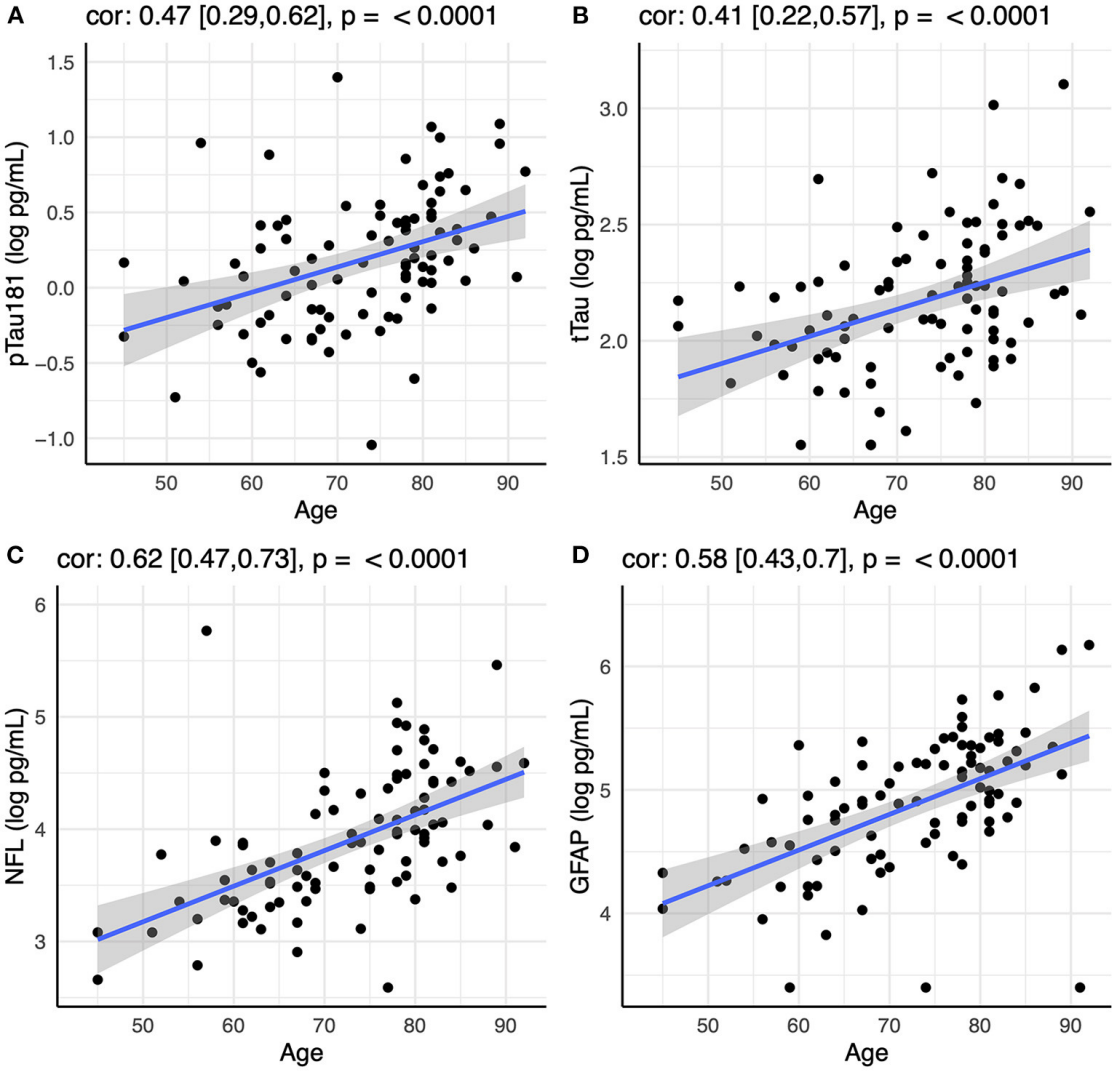
Correlation between age and plasma levels of **(A)** pTau181, **(B)** tTau, **(C)** NfL, and **(D)** GFAP using Spearman rank-order correlation. Spearman's rho with 95% confidence interval and *p*-values are show on top of each panel.

Initial analysis showed that all four biomarkers (pTau181, tTau, NfL, and GFAP) increased with age in the CN group (Figure 1). There was also an effect of sex for pTau181 in the CN group, with males having higher pTau181 levels than females (*p* < 0.003). This was not observed within the AD or OND groups and was attenuated in the CN group by controlling for age (*p* < 0.02). All subsequent analyses were controlled for age and sex.

### 3.3. Correlations among AD biomarkers

pTau181 and tTau were strongly correlated not only among individuals with AD (*r* = 0.54, *p* < 0.001), but also among CN and ONDs (*r* = 0.55 and 0.58, respectively; *p* < 0.001). pTau181 and tTau correlated moderately with GFAP (GFAP/pTau181: *r* = 0.35, *p* < 0.001; GFAP/tTau: *r* = 0.30, *p* < 0.005) and NfL (NfL/pTau181: *r* = 0.35, *p* < 0.001; NFL/tTau: *r* = 0.54, *p* < 0.001) within the AD group, but these correlations were lost among ONDs (GFAP/pTau181: *r* = 0.08; GFAP/tTau: *r* = 0.10; NfL/pTau181: *r* = 0.06; NfL/tTau: *r* = 0.32), likely reflecting that disease mechanisms other than amyloid and tau pathology also increase GFAP and NfL levels in these individuals.

### 3.4. Plasma pTau181 and GFAP can predict cognitive decline in participants with MCI

**Figure 2:**
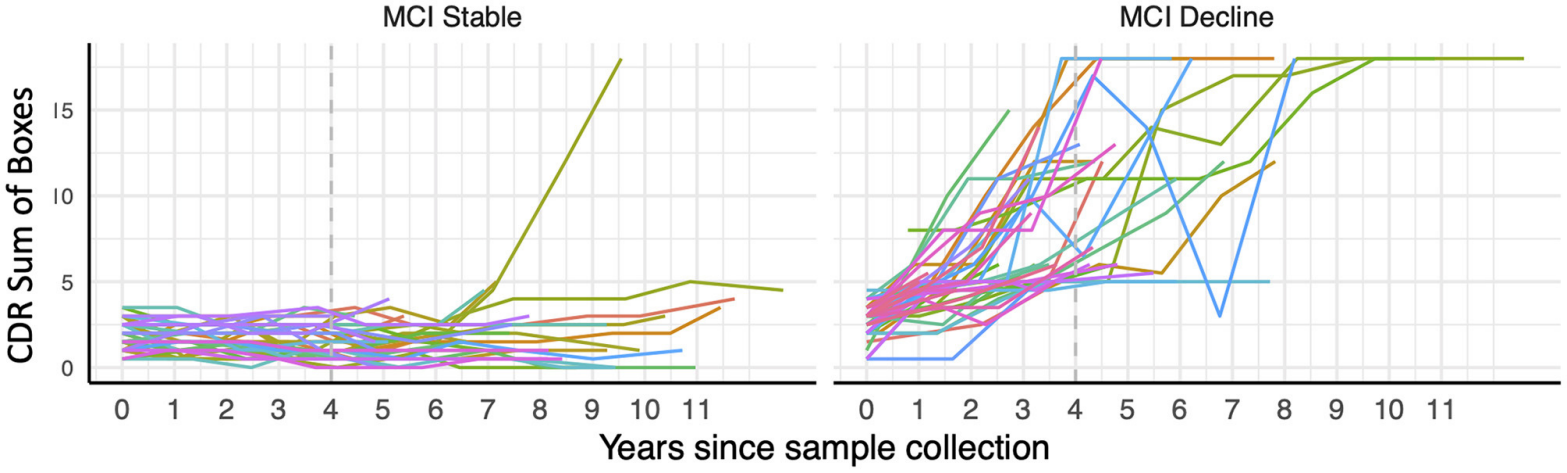
Trajectory of CDR Sum of Boxes scores over longitudinal visits in participants with mild cognitive impairment (MCI) at the time of blood draw classified as stable or decliners based on their cognitive trajectories during 4 years of follow-up (indicated with dashed gray line).

**Figure 3:**
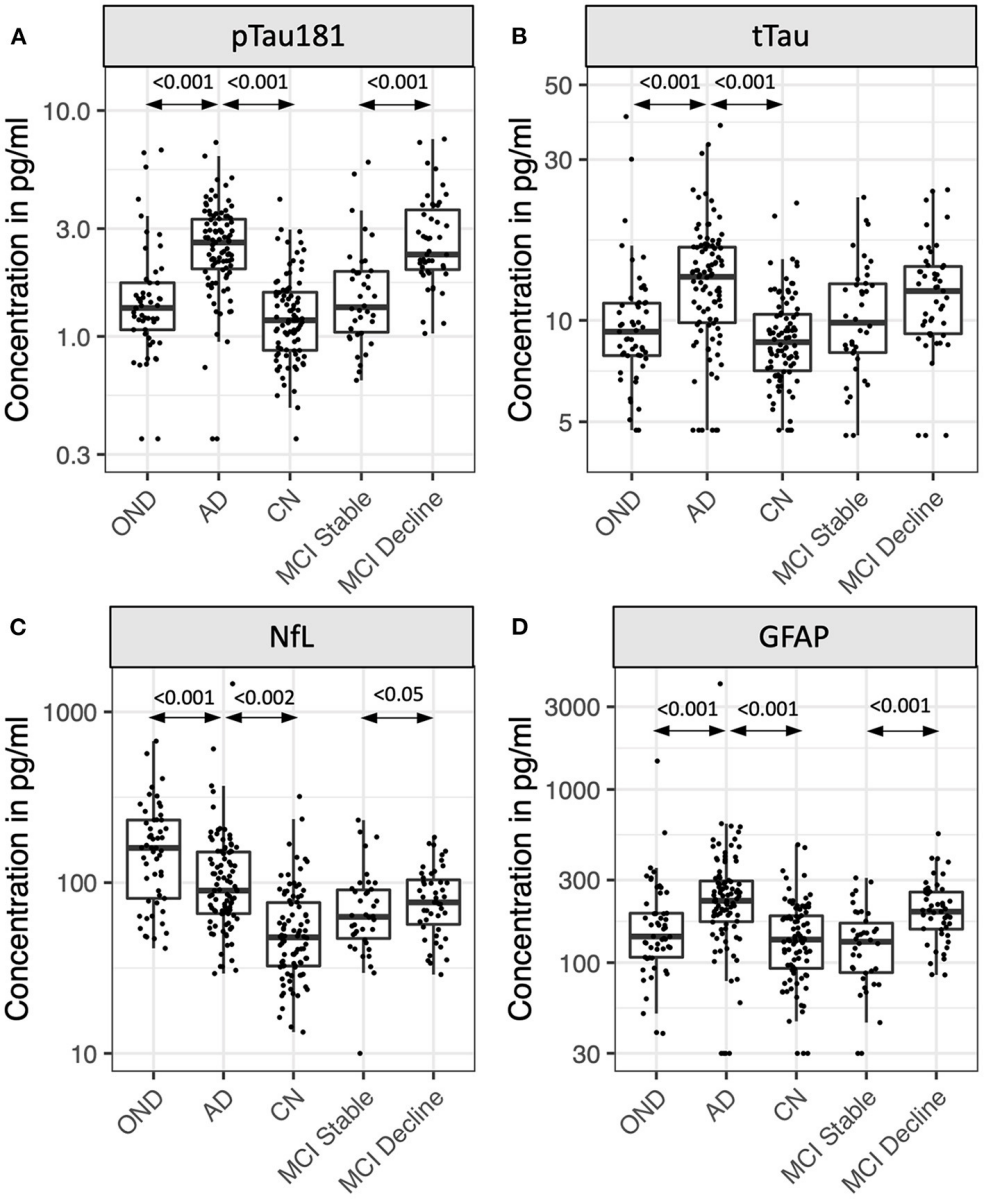
Plasma levels of pTau181 **(A)**, tTau **(B)**, NfL **(C)**, and GFAP **(D)** in participants with Alzheimer's disease (AD), other neurodegenerative diseases (OND), normal cognition (CN), and mild cognitive impairment (MCI). MCI participants were stratified by the absence (stable) or presence (decline) of cognitive decline during 4 years of follow-up. Box plots show median, 25th/75th percentile, and smallest/largest value within 1.5 × the interquartile below/above the median. Select *p*-values are indicated in graph.

**Figure 4:**
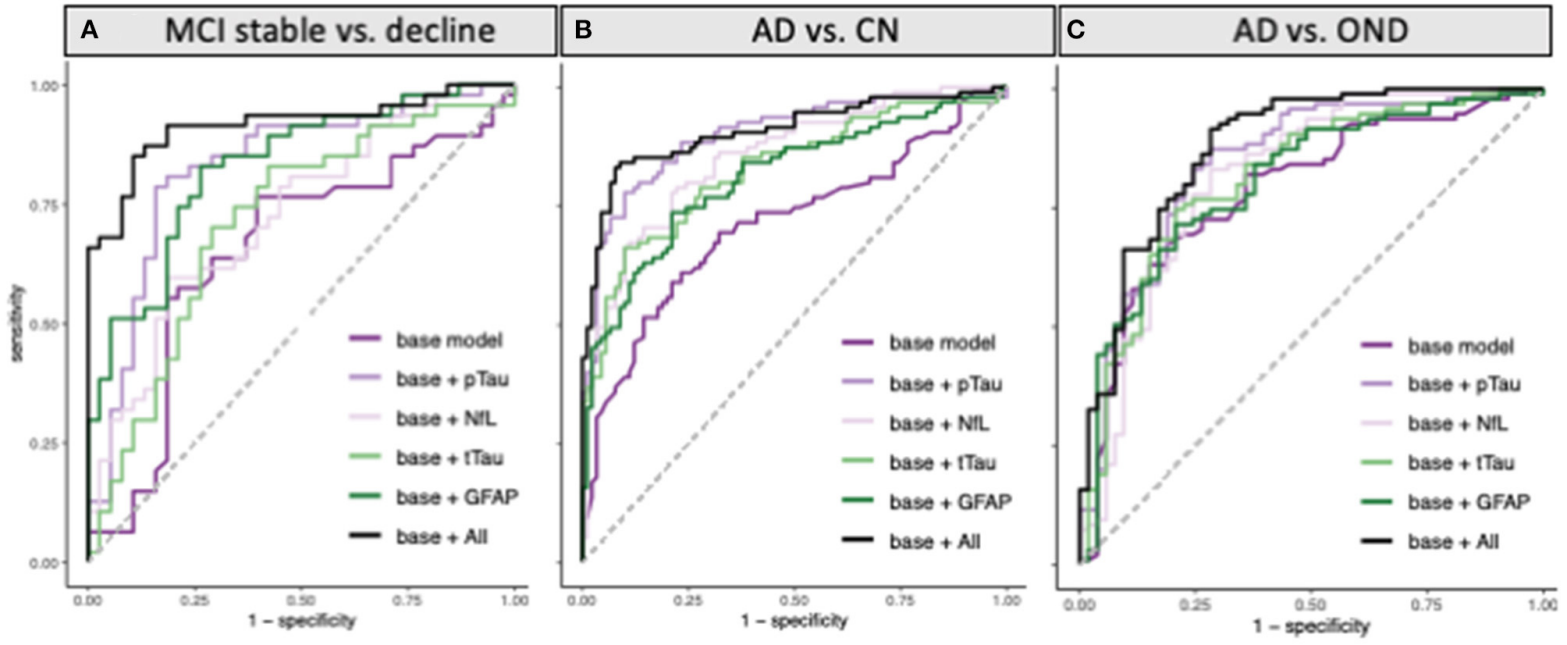
ROC curves for classification of **(A)** MCI participants who progressed to a consensus diagnosis of AD dementia during 4 years of follow-up (MCI decline) vs. participants who remained stable during follow-up (MCI stable). **(B)** AD vs. cognitively normal controls (CN), and **(C)** AD vs. other neurodegenerative diseases (OND) using pTau181, tTau, NfL, and GFAP alone or all combined in addition to a base model containing age, sex, and APOE status.

Eighty five participants with MCI at baseline and 4 years of follow-up (Table 2, Figure 2) were investigated to determine if plasma biomarker levels at baseline can predict clinical progression. MCI-decline participants who progressed to a consensus diagnosis of AD dementia during follow up had higher baseline plasma concentrations of pTau181, NfL, and GFAP compared to MCI-stable participants with the largest fold change for pTau181 and GFAP (1.7 and 1.6-fold increase, respectively; *p* < 0.001 for both comparisons; Figure 3, Table 3). NfL levels were significantly higher in MCI-decline (*p* < 0.05) compared to stable participants, but the difference was modest (1.1-fold increase). Adding either pTau181 or GFAP to a logistic regression model including age, sex, and APOE status increased the ability to discriminate between MCI-decline and MCI-stable participants from an AUC of 0.65 (CI: 0.56–0.78) to an AUC of 0.83 (CI: 0.74–0.92; likelihood ratio test *p* < 0.001; Figure 4, Table 3). The combination of GFAP and pTau181 further improved the ability to predict progression (AUC = 0.89; CI 0.83–0.96; *p* < 0.001), while adding also tTau and NfL only increased the ability to predict progression marginally (AUC = 0.92; CI: 0.85–0.98).

**Table 2:** Demographic and clinical information.

	**MCI_Stable**	**MCI_Decline**	***p*-value**	**CN**	**AD**	**OND^a^**	***p*-value**
*n* (% Female)	38 (44.7%)	47 (48.9%)	n.s.	90 (56.7%)	95 (47.4%)	53 (45.3%)	n.s.
Non-Hispanic white, n (%)	35 (92.1)	45 (95.7)	n.s.	79 (87.8%)	93 (97.9%)	51 (96.2%)	<0.02
Age (mean ± SD)	77.8 ± 7.4	75.7 ± 8.6	n.s.	72.6 ± 10.4	74.2 ± 10.6	69.4 ± 10.9	<0.05
CDR, Global (mean ± SD)	0.50 ± 0.00	0.50 ± 0.00	n.s.	0.00 ± 0.00	1.28 ± 0.89	1.18 ± 0.88	<0.001
CDR Sum of Boxes (mean ± SD)	1.87 ± 0.87	2.65 ± 0.89	<0.001	0.00 ± 0.00	7.16 ± 5.35	6.38 ± 5.47	<0.001
MMSE score (mean ± SD)	28.29 ± 1.43	26.12 ± 4.23	<0.005	29.55 ± 0.75	17.61 ± 9.02	24.62 ± 5.93	<0.001

MCI, mild cognitive decline; CN, cognitive normal controls; AD, Alzheimer's Disease; OND, other neurodegenerative diseases; CDR, Clinical Dementia Rating; MMSE, Mini mental state examination.

^a^Frontotemporal Lobar Degeneration (FTLD) TDP43 (*n* = 13), FTLD tau (*n* = 19), Lewy body disease (*n* = 7), cerebrovascular disease (*n* = 5), amyotrophic lateral sclerosis (*n* = 4), Creutzfeldt-Jakob disease (*n* = 1), cerebral amyloid angiopathy (*n* = 1), multiple sclerosis (*n* = 1), thalamic degeneration (*n* = 1), and dementia lacking distinctive histology (*n* = 1).

**Table 3:** Clinical performance of the four biomarker assays in Sample A (Longitudinal prognostic sample).

	**MCI Decline (*****n*** = **47)**		**MCI Stable (*****n*** = **38)**				
	**Mean (SD)**	**Median (Q1-Q3)**	**Mean (SD)**	**Median (Q1-Q3)**	**Fold change**	**Cohen's d**	***p*** **-value**
pTau181 (pg/mL)	2.88 (1.44)	2.31 (1.94–3.80)	1.73 (1.13)	1.35 (1.03–1.95)	1.67	1.15	<0.001
tTau (pg/mL)	12.5 (4.6)	12.2 (9.1–14.5)	10.8 (4.3)	9.8 (8.0–12.9)	1.15	0.39	n.s.
NfL (pg/mL)	85 (38.4)	76.7 (55–106.6)	76.7 (47.8)	63 (46.3–91.4)	1.11	0.32	<0.05
GFAP (pg/mL)	213 (93)	197 (155–257)	137 (69)	132 (87–174)	1.56	1.03	<0.001
	**Differentiation MCI stable vs. decline**						
	**AUC (95% CI)**		**%Sensitivity**		**%Specificity**		
Base model (age, sex, APOE)	0.65 (0.53–0.78)		77%		61%		
Base model + pTau181	0.83 (0.74–0.92)		79%		84%		
Base model + tTau	0.72 (0.60–0.83)		70%		71%		
Base model + NfL	0.73 (0.62–0.83)		60%		82%		
Base model + GFAP	0.83 (0.74–0.92)		83%		74%		
Base model + all four biomarkers	0.92 (0.85–0.98)		85%		89%		

MCI, mild cognitive impairment; Q1/Q3, quartile 1/3; AUC, area under the curve.

Reported sensitivity and specificity were determined at the point of maximum sensitivity and specificity determined by the Youden index.

### 3.5. Plasma biomarker levels can differentiate AD from controls

The diagnoses of the MCI participants were based solely on clinical presentation so we next assessed if plasma biomarker levels could differentiate participants with autopsy- or biomarker verified AD from controls with OND or CN adults in a cross-sectional sample (Table 2) to understand if pTau181 and GFAP identified MCI participants with AD as underlying pathology or if their levels predicted disease progression *per se*.

Between group differences in plasma biomarker concentrations demonstrated that participants with AD had roughly 2-fold higher plasma concentrations of pTau181, NfL, and GFAP compared to CN, while tTau concentrations on average were 1.5-fold higher in AD (*p* < 0.001 for all comparisons; Figure 3, Table 4). Adding pTau181 to a logistic regression model including age, sex, and APOE increased the discrimination between AD and CN from an AUC of 0.71 to 0.90 (*p* < 0.001; Figure 4) with 78% sensitivity and 90% specificity (Table 4). Corresponding AUCs for tTau, NfL, and GFAP ranged between 0.81 and 0.83 (*p* < 0.001 for all analytes) and adding all three to the base model plus pTau181 did not increase the ability to discriminate between AD and CN compared to the model with pTau181 (AUC = 0.91; CI: 0.86–0.95).

**Table 4:** Clinical performance of the four biomarker assays in Sample B (“High contrast” diagnostic sample).

	**CN (*****n*** = **90)**		**AD (*****n*** = **95)**		**OND (*****n*** = **53)**	
	**Mean (SD)**	**Median (Q1–Q3)**	**Mean (SD)**	**Median (Q1–Q3)**	**Mean (SD)**	**Median (Q1–Q3)**
pTau181 (pg/mL)	1.32 ± 0.63	1.18 (0.87–1.57)	2.67 ± 1.13	2.61 (1.97–3.32)	1.72 ± 1.33	1.34 (1.07–1.73)
tTau (pg/mL)	9.2 ± 3.1	8.6 (7.0–10.4)	13.7 ± 5.8	13.5 (9.8–16.5)	10.4 ± 5.9	9.3 (7.9–11.2)
NfL (pg/mL)	59.9 ± 46.2	47.8 (32.4–76.9)	127.4 ± 160.0	89.9 (65.8–152.0)	178.2 ± 126.4	159.6 (80.9–232.6)
GFAP (pg/mL)	151 ± 82	136 (92–187)	293 ± 413	228 (172–299)	194 ± 201	142 (108–193)
	**AD vs. CN**			**AD vs. OND**			**CN vs. OND**		
	**Fold change**	**Cohen's d**	***p*** **-value**	**Fold change**	**Cohen's d**	***p*** **-value**	**Fold change**	**Cohen's d**	***p*** **-value**
pTau181 (pg/mL)	2.02	1.50	<0.001	1.55	1.01	<0.001	1.30	0.34	n.s.
tTau (pg/mL)	1.50	0.99	<0.001	1.31	0.67	<0.001	1.14	0.25	n.s.
NfL (pg/mL)	2.13	1.10	<0.001	0.71	0.58	<0.002	2.97	1.68	<0.001
GFAP (pg/mL)	1.94	0.83	<0.001	1.51	0.54	<0.005	1.29	0.28	n.s.
	**Differentiation AD vs. CN**			**Differentiation AD vs. OND**					
	**AUC (95% CI)**	**%Sens**	**%Spec**	**AUC (95% CI)**	**%Sens**	**%Spec**			
Base model (age, sex, APOE)	0.71 (0.64–0.79)	59%	79%	0.79 (0.71–0.86)	67%	81%			
Base model + pTau181	0.90 (0.85–0.94)	78%	90%	0.84 (0.77–0.91)	87%	72%			
Base model + tTau	0.83 (0.77–0.89)	66%	90%	0.81 (0.74–0.89)	83%	72%			
Base model + NfL	0.85 (0.80–0.91)	78%	79%	0.81 (0.74–0.89)	72%	79%			
Base model + GFAP	0.81 (0.75–0.87)	74%	79%	0.80 (0.73–0.88)	75%	79%			
Base model + all four biomarkers	0.91 (0.86–0.95)	83%	92%	0.88 (0.82-0.94)	92%	72%			

CN, cognitively normal; AD, Alzheimer's Disease; OND, other neurodegenerative diseases; Q1/Q3, Quartile 1/3; AUC, area under the curve. Reported sensitivity (sens) and specificity (spec) were determined at the point of maximum sensitivity and specificity determined by the Youden index.

Participants with AD also had roughly 1.3–1.5-fold higher plasma pTau181, tTau, and GFAP concentrations than ONDs (pTau181 and tTau: *p* < 0.001; GFAP: *p* < 0.005; Figure 3, Table 4), again with pTau181 showing the largest fold difference between the groups. NfL concentrations were, in contrast, 1.4-fold higher in ONDs compared to AD (*p* < 0.001), consistent with it being a non-specific marker for neuronal injury (18). Adding pTau181 to a logistic regression model with age, sex, and APOE increased the AUCs for differentiating between AD and OND from 0.79 (CI: 0.71–0.86) to 0.84 (0.77–0.91; *p* < 0.001) while adding tTau, NfL, or GFAP resulted in a marginally increased AUC of 0.80–0.81 (Figure 4, Table 4). Adding all four biomarkers to the base model increased the AUC to 0.88 (0.82–0.94; *p* < 0.001).

Next, we calculated an optimal pTau181 threshold to differentiate AD from CN using the Youden index and applied this threshold to the MCI sample. This demonstrated that the majority of MCI-decline participants (39/47; 83%) had pTau181 levels consistent with AD at baseline, while only a minority of the MCI-stable group (9/38; 24%; *p* < 0.001) had such high levels. In contrast, pTau181 levels at baseline did not correlate with rate of disease progression calculated as a linear estimate of the increase in CDR SOB over the 4 years of follow-up when limiting the analysis to MCI participants classified as AD using the pTau181 threshold (data not shown).

### 3.6. Cross-sectional correlation with disease severity and cognitive function

**Figure 5:**
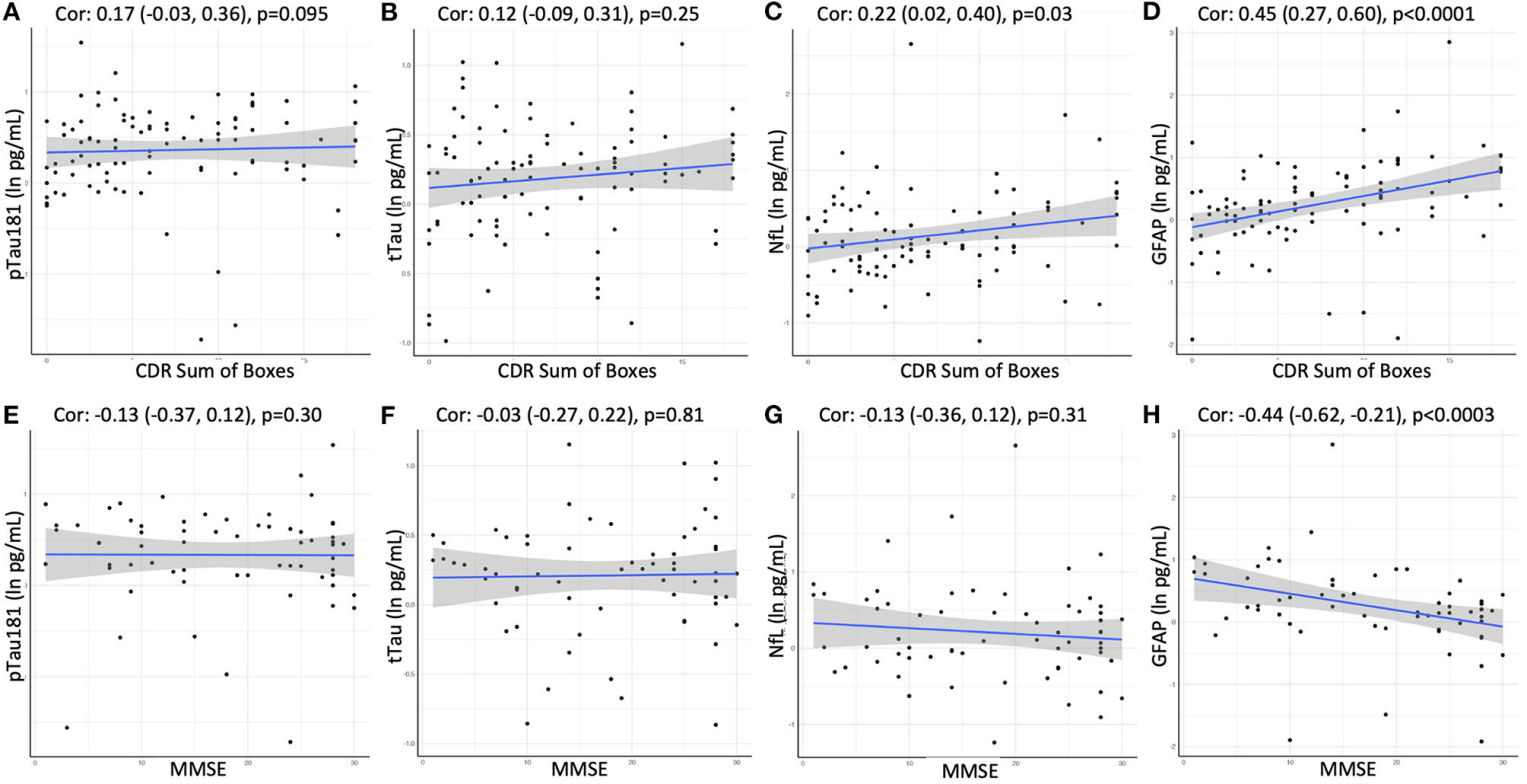
Correlation between CDR Sum of Boxes or MMSE and plasma levels of **(A, E)** pTau181, **(B, F)** tTau, **(C, G)** NfL, and **(D, H)** GFAP using Spearman rank-order correlation. Spearman's rho with 95% confidence interval and *p*-values are show on top of each panel.

Finally, we assessed if plasma biomarker concentrations were significantly associated with disease severity or global cognitive function at the time of the blood draw in the cross-sectional sample. Clinical dementia severity was assessed by global CDR and CDR sum of boxes (SOB) scores in all participants. Cognitive impairment was evaluated using the Mini-Mental State Examination in 64 participants. A positive association in this analysis was observed for GFAP, which showed moderate correlations with global CDR (Spearman's rho = 0.44; *p* < 0.001), CDR SOB (rho = 0.45; *p* < 0.001; Figure 5), as well as MMSE (rho = −0.44; *p* < 0.001; Figure 5). We also observed weak correlations between NfL and CDR SOB (rho = 0.22; *p* < 0.05) as well as global CDR (rho = 0.19; *p* < 0.06), while no associations were observed for pTau181 or tTau.

## 4. Discussion

We describe the diagnostic and prognostic value of four plasma biomarkers of AD neuropathology in 307 participants in the longitudinal cohort of the Massachusetts ADRC. We confirm previous findings that all four biomarkers provide predictive diagnostic value for AD, and we extend emerging findings that pTau181, GFAP and to a lesser degree NfL inform prognosis with their higher levels predicting decline in participants with MCI.

pTau181, tTau, NfL, and GFAP were measured in plasma using novel ECL-based MSD^®^ immunoassays. The assays, developed using the ultrasensitive S-PLEX ECL assay format (19–22), performed well and detected higher plasma levels of all four biomarkers in individuals with AD compared to both individuals with normal cognition and, with the exception of NfL, individuals with other non-AD neurodegenerative diseases. The best performance was observed for pTau181, which could discriminate between AD and CN with an AUC of 0.90 and between AD and non-AD neurodegenerative diseases with an AUC of 0.84. This diagnostic accuracy between AD and CN is comparable to that originally observed using SIMOA assays on the Quanterix platform and what subsequently has been reported in several studies (3, 4, 6, 23, 24). The diagnostic performance of the pTau181 S-PLEX assay was notably better in our study than in a recent head-to-head comparison of several pTau181 and−217 assays for differentiating amyloid-β PET positive and negative individuals with MCI (25). We also observed that pTau181 measured using the S-PLEX assay could predict clinical decline in individuals with MCI with good accuracy, comparable to the performance of the best pTau181 assays in that study. We speculate that the discrepancies between studies may be due to differences in study populations, including a broader range of severity in our study, many with autopsy confirmation of disease, differences in definitions of MCI, and lengths of follow-up. There also may be site-specific differences in technical performance. Further round robin studies with larger numbers of identical samples containing more diverse patient populations measured using multiple assays performed at different sites would be useful.

In line with an emerging literature showing prognostic utility of plasma biomarkers (6, 8–10), we observed that both pTau181 and GFAP had good accuracy in predicting clinical decline in individuals with MCI. These participants did not have imaging or CSF biomarker data available to definitively establish an AD diagnosis, resembling the situation in routine clinical practice where a central question often is what the likelihood is for further cognitive decline. It has been suggested that individuals with MCI who progress to dementia have a clinical profile typical of AD while stable MCI more frequently is caused by other underlying morbidities such as vascular disease (26), prompting us to ask if pTau181 discriminates between MCI due to AD vs. MCI due to other causes. Using the pTau181 threshold established for AD in the diagnostic sample, we noted that significantly more MCI decline participants had pTau181 levels consistent with AD compared to the MCI stable group while we failed to see a correlation between baseline pTau181 levels and rate of decline as measured by CDR SOB in MCI participants with pTau181 levels consistent with AD. Taken together this suggest that the ability of pTau181 to predict decline in this MCI sample is more related to the underlying AD pathology than AD or other neurodegenerative dementia progression *per se* with the caveat that a larger sample size or more advanced modeling of progression may be required to identify a correlation between pTau181 levels and cognitive decline. Nevertheless, the data provide evidence for the prognostic use of plasma biomarkers in MCI in order to infer presumed AD pathology.

The sensitivity of ECL assays has not previously been adequate to quantify plasma NfL levels in AD (27), but advances in ultrasensitive detection in the ECL assays using additional signal enhancement (19) have made it possible to detect levels in the single picogram range using the current enhanced assay. Notably, while the vast majority of other assays use the “gold standard” antibody pair developed by Uman Diagnostics (28), the current assay uses a novel antibody pair developed by MSD.

Increased plasma levels of tTau have previously been described in AD, but differences in average levels between AD and control groups have been small, their distributions largely overlapping, and plasma tTau levels were only weakly correlated with CSF tTau limiting the usefulness of plasma tTau as measured in that assay as a diagnostic marker in AD (29–31). The novel ECL assay tested here showed better separation between the AD and CN groups than previous studies, with 1.5-fold higher average concentrations in AD compared to CN and a diagnostic accuracy of 0.83. It is thought that the discrepancy in tTau levels between CSF and plasma may be explained by proteolytic degradation of tau in the blood or by contribution of peripheral tau (32). We observed a strong correlation between tTau and pTau181 levels using the ECL assays in our study suggesting that the observed tTau levels do, at least in part, reflect AD pathology. It can be speculated that the epitopes detected by the current assay detect tau fragments that are more stable in plasma, by analogy to the recently described N-terminal tau fragment NT-1, which similarly was highly predictive of future cognitive decline and pathological tau accumulation in clinically normal elderly (33). The current data reinforce the potential for tau-based plasma biomarkers to have utility in prediction of cognitive decline in patients with mild impairments.

The strengths of this study include a well characterized diagnostic sample with autopsy- or biomarker verified diagnosis of the participants with AD and non-AD neurodegenerative diseases, and the careful selection of individuals with at least 4 years of follow up for the prognostic analyses. Adequate sample sizes were used and the groups were similar in age and other demographic attributes. Initial analysis indicated an effect of age on biomarker levels and all analysis was therefore controlled for age. Limitations include that some of the CN participants in the diagnostic sample may have been misclassified due to the lack of molecular verification of the diagnosis. It is furthermore possible that comorbid pathologies (i.e., vascular, Lewy body, TDP-43) may contribute to the progression in the MCI sample but could not be accounted for due to the lack of autopsy confirmation or relevant biomarkers. Lastly, our study population consisted largely of white non-Hispanics, which limits the generalizability of the results.

The new generation of ultrasensitive ECL assays evaluated in this study provided sufficient accuracy to serve both as diagnostic and prognostic biomarkers in AD and can be measured using technology currently widely available in research laboratories. The rapid development of ultrasensitive assays for measuring AD biomarkers in blood holds promise to transform clinical practice and clinical research in providing affordable and easily accessible assays to assist in diagnosis and prognosis that can be implemented not only in large, centralized settings but equally well in community settings and smaller laboratories lacking the resources to procure expensive specialized equipment. While more research is needed to determine optimal combinations of AD biomarkers and assays, their diagnostic thresholds, and their accuracy in detecting AD pathology in heterogeneous patient populations with low AD prevalence and frequent comorbidities before blood biomarkers can be used in clinical settings, the recent Alzheimer's Association appropriate use recommendations for blood biomarkers in AD suggest that blood biomarkers including pTau can be used as a first screening step in clinical trials and to exclude patients with AD co-pathology from non-AD trials (34). Identifying individuals with MCI with high likelihood of progressing to AD dementia will be an important step in screening individuals for inclusion in clinical trials as well as for future therapeutic interventions.

## Data availability statement

The raw data supporting the conclusions of this article will be made available by the authors, without undue reservation.

## Ethics statement

The studies involving human participants were reviewed and approved by Mass General Brigham Internal Review Board. The patients/participants provided their written informed consent to participate in this study.

## Author contributions

Study concept and design: PK, BC, GS, MS, and SA. Acquisition, analysis or interpretation of data, and critical revision of the manuscript for important intellectual content: PK, BC, TS, BT, KL, LE-M, IT, LC, CS, KJ, BD, TG-I, DB, DO, MF, BH, AA, PB, CC, GS, MS, and SA. Drafting of manuscript: PK. Statistical analysis: TS and LC. Obtained funding: CS, BH, GS, MS, and SA. Study supervision: PK, BC, MS, and SA. All authors contributed to the article and approved the submitted version.

## References

[1] Jack CR Bennett DA Blennow K Carrillo MC Dunn B Haeberlein SB. NIA-AA Research Framework: Toward a biological definition of Alzheimer's disease. Alzheimers Dement. (2018) 14:535–62. 10.1016/j.jalz.2018.02.018 29653606 PMC5958625

[2] Leuzy A Mattsson-Carlgren N Palmqvist S Janelidze S Dage JL Hansson O. Blood-based biomarkers for Alzheimer's disease. EMBO Mol Med. (2021) 2–6:e14408. 10.15252/emmm.202114408 34859598 PMC8749476

[3] Bayoumy S Verberk IMW den Dulk B Hussainali Z Zwan M van der Flier WM. Clinical and analytical comparison of six Simoa assays for plasma P-tau isoforms P-tau181, P-tau217, and P-tau231. Alzheimers Res Ther. (2021) 13:198. 10.1186/s13195-021-00939-9 34863295 PMC8645090

[4] Karikari TK Pascoal TA Ashton NJ Janelidze S Benedet AL Rodriguez JL. Blood phosphorylated tau 181 as a biomarker for Alzheimer's disease: a diagnostic performance and prediction modelling study using data from four prospective cohorts. Lancet Neurol. (2020) 19:422–33. 10.1016/S1474-4422(20)30071-5 32333900

[5] Palmqvist S Janelidze S Quiroz YT Zetterberg H Lopera F Stomrud E. Discriminative accuracy of plasma phospho-tau217 for Alzheimer disease vs other neurodegenerative disorders. JAMA. (2020) 324:772–81. 10.1001/jama.2020.12134 32722745 PMC7388060

[6] Janelidze S Mattsson N Palmqvist S Smith R Beach TG Serrano GE. Plasma P-tau181 in Alzheimer's disease: relationship to other biomarkers, differential diagnosis, neuropathology and longitudinal progression to Alzheimer's dementia. Nat Med. (2020) 26:379–86. 10.1038/s41591-020-0755-1 32123385

[7] Thijssen EH La Joie R Strom A Fonseca C Iaccarino L Wolf A. Plasma phosphorylated tau 217 and phosphorylated tau 181 as biomarkers in Alzheimer's disease and frontotemporal lobar degeneration: a retrospective diagnostic performance study. Lancet Neurol. (2021) 20:739–52. 10.1016/S1474-4422(21)00214-3 34418401 PMC8711249

[8] Therriault J Benedet AL Pascoal TA Lussier FZ Tissot C Karikari TK. Association of plasma P-tau181 with memory decline in non-demented adults. Brain Commun. (2021) 3:fcab136. 10.1093/braincomms/fcab136 34222875 PMC8249102

[9] Karikari TK Benedet AL Ashton NJ Lantero Rodriguez J Snellman A Suarez-Calvet M. Diagnostic performance and prediction of clinical progression of plasma phospho-tau181 in the Alzheimer's Disease Neuroimaging Initiative. Mol Psychiatry. (2021) 26:429–42. 10.1038/s41380-020-00923-z 33106600

[10] Wang YL Chen J Du ZL Weng H Zhang Y Li R. Plasma p-tau181 level predicts neurodegeneration and progression to Alzheimer's dementia: a longitudinal study. Front Neurol. (2021) 12:695696. 10.3389/fneur.2021.695696 34557143 PMC8452983

[11] Besser L Kukull W Knopman DS Chui H Galasko D Weintraub S. Version 3 of the national Alzheimer's coordinating center's uniform data set. Alzheimer Dis Assoc Disord. (2018) 32:351–8. 10.1097/WAD.0000000000000279 30376508 PMC6249084

[12] Weintraub S Besser L Dodge HH Teylan M Ferris S Goldstein FC. Version 3 of the Alzheimer disease centers' neuropsychological test battery in the uniform data set (UDS). Alzheimer Dis Assoc Disord. (2018) 32:10–7. 10.1097/WAD.0000000000000223 29240561 PMC5821520

[13] Albert MS DeKosky ST Dickson D Dubois B Feldman HH Fox NC. The diagnosis of mild cognitive impairment due to Alzheimer's disease: recommendations from the National Institute on Aging-Alzheimer's Association workgroups on diagnostic guidelines for Alzheimer's disease. Alzheimers Dement. (2011) 7:270–9. 10.1016/j.jalz.2011.03.008 21514249 PMC3312027

[14] McKhann GM Knopman DS Chertkow H Hyman BT Jack CR Jr Kawas CH. The diagnosis of dementia due to Alzheimer's disease: recommendations from the National Institute on Aging-Alzheimer's Association workgroups on diagnostic guidelines for Alzheimer's disease. Alzheimers Dement. (2011) 7:263–9. 10.1016/j.jalz.2011.03.005 21514250 PMC3312024

[15] Montine TJ Phelps CH Beach TG Bigio EH Cairns NJ Dickson DW. National Institute on Aging-Alzheimer's Association guidelines for the neuropathologic assessment of Alzheimer's disease: a practical approach. Acta Neuropathol. (2012) 123:1–11. 10.1007/s00401-011-0910-3 22101365 PMC3268003

[16] Mohammadi D. The Harvard Biomarker Study's big plan. Lancet Neurol. (2013) 12:739–40. 10.1016/S1474-4422(13)70155-8 23809962

[17] Robin X Turck N Hainard A Tiberti N Lisacek F Sanchez JC. pROC: an open-source package for R and S+ to analyze and compare ROC curves. BMC Bioinformatics. (2011) 12:77. 10.1186/1471-2105-12-77 21414208 PMC3068975

[18] Yuan A Nixon RA. Neurofilament proteins as biomarkers to monitor neurological diseases and the efficacy of therapies. Front Neurosci. (2021) 15:689938. 10.3389/fnins.2021.689938 34646114 PMC8503617

[19] Broger T Tsionksy M Mathew A Lowary TL Pinter A Plisova T. Sensitive electrochemiluminescence (ECL) immunoassays for detecting lipoarabinomannan (LAM) and ESAT-6 in urine and serum from tuberculosis patients. PLoS ONE. (2019) 14:e0215443. 10.1371/journal.pone.0215443 30998715 PMC6472883

[20] Diamandis EP Stanczyk FZ Wheeler S Mathew A Stengelin M Nikolenko G. Serum complexed and free prostate-specific antigen (PSA) for the diagnosis of the polycystic ovarian syndrome (PCOS). Clin Chem Lab Med. (2017) 55:1789–97. 10.1515/cclm-2016-1124 28361781 PMC5742006

[21] Poorbaugh J Samanta T Bright SW Sissons SE Chang CY Oberoi P. Measurement of IL-21 in human serum and plasma using ultrasensitive MSD S-PLEX(R) and Quanterix SiMoA methodologies. J Immunol Methods. (2019) 466:9–16. 10.1016/j.jim.2018.12.005 30590020

[22] Pollock NR Savage TJ Wardell H Lee RA Mathew A Stengelin M. Correlation of SARS-CoV-2 nucleocapsid antigen and RNA concentrations in nasopharyngeal samples from children and adults using an ultrasensitive and quantitative antigen assay. J Clin Microbiol. (2021) 59:e03077–20. 10.1128/JCM.03077-20 33441395 PMC8092747

[23] Simren J Leuzy A Karikari TK Hye A Benedet AL Lantero-Rodriguez J. The diagnostic and prognostic capabilities of plasma biomarkers in Alzheimer's disease. Alzheimers Dement. (2021) 17:1145–56. 10.1002/alz.12283 33491853 PMC8359457

[24] Mielke MM Hagen CE Xu J Chai X Vemuri P Lowe VJ. Plasma phospho-tau181 increases with Alzheimer's disease clinical severity and is associated with tau- and amyloid-positron emission tomography. Alzheimers Dement. (2018) 14:989–97. 10.1016/j.jalz.2018.02.013 29626426 PMC6097897

[25] Janelidze S Bali D Ashton NJ Barthelemy NR Vanbrabant J Stoops E. Head-to-head comparison of 10 plasma phospho-tau assays in prodromal Alzheimer's disease. Brain. (2022). 10.1093/brain/awac333 [Epub ahead of print]. 36087307 PMC10115176

[26] Ganguli M Jia Y Hughes TF Snitz BE Chang CH Berman SB. Mild cognitive impairment that does not progress to dementia: a population-based study. J Am Geriatr Soc. (2019) 67:232–8. 10.1111/jgs.15642 30444944 PMC6367026

[27] Li D Mielke MM. An update on blood-based markers of Alzheimer's disease using the SiMoA platform. Neurol Ther. (2019) 8:73–82. 10.1007/s40120-019-00164-5 31833025 PMC6908531

[28] Forgrave LM Ma M Best JR DeMarco ML. The diagnostic performance of neurofilament light chain in CSF and blood for Alzheimer's disease, frontotemporal dementia, and amyotrophic lateral sclerosis: a systematic review and meta-analysis. Alzheimers Dement. (2019) 11:730–43. 10.1016/j.dadm.2019.08.009 31909174 PMC6939029

[29] Dage JL Wennberg AMV Airey DC Hagen CE Knopman DS Machulda MM. Levels of tau protein in plasma are associated with neurodegeneration and cognitive function in a population-based elderly cohort. Alzheimers Dement. (2016) 12:1226–34. 10.1016/j.jalz.2016.06.001 27436677 PMC5148697

[30] Mattsson N Zetterberg H Janelidze S Insel PS Andreasson U Stomrud E. Plasma tau in Alzheimer disease. Neurology. (2016) 87:1827–35. 10.1212/WNL.0000000000003246 27694257 PMC5089525

[31] Zetterberg H Wilson D Andreasson U Minthon L Blennow K Randall J. Plasma tau levels in Alzheimer's disease. Alzheimers Res Ther. (2013) 5:9. 10.1186/alzrt163 23551972 PMC3707015

[32] Zetterberg H Blennow K. Moving fluid biomarkers for Alzheimer's disease from research tools to routine clinical diagnostics. Mol Neurodegener. (2021) 16:10. 10.1186/s13024-021-00430-x 33608044 PMC7893769

[33] Chhatwal JP Schultz AP Dang Y Ostaszewski B Liu L Yang HS. Plasma N-terminal tau fragment levels predict future cognitive decline and neurodegeneration in healthy elderly individuals. Nat Commun. (2020) 11:6024. 10.1038/s41467-020-19543-w 33247134 PMC7695712

[34] Hansson O Edelmayer RM Boxer AL Carrillo MC Mielke MM Rabinovici GD. The Alzheimer's Association appropriate use recommendations for blood biomarkers in Alzheimer's disease. Alzheimers Dement. (2022). 10.1002/alz.070020 35908251 PMC10087669

